# Needle beam two-photon microscopy for simultaneous multiplane neural and vascular imaging in awake mice

**DOI:** 10.1186/s43074-026-00237-3

**Published:** 2026-03-16

**Authors:** Quanyu Zhou, Jingjing Zhao, Chaim Glück, Yu-Hang Liu, Lin Du, Lukas Glandorf, Tian Jin, Zhenyue Chen, Lingqi Jiang, Bruno Weber, Adam de la Zerda, Daniel Razansky

**Affiliations:** 1 Institute for Biomedical Engineering and Institute of Pharmacology and Toxicology, Faculty of Medicine, University of Zurich, Zurich, Switzerland; 2 Institute for Biomedical Engineering, Department of Information Technology and Electrical Engineering, ETH Zurich, Zurich, Switzerland; 3 School of Medical Equipment Science and Engineering, Huazhong University of Science and Technology, Wuhan, China; 4 College of Life Science and Technology, Huazhong University of Science and Technology, Wuhan, China; 5 Department of Structural Biology, Stanford University School of Medicine, Stanford University, Stanford, USA; 6 Biophysics Program, Molecular Imaging Program, and Bio-X Program at Stanford University, Stanford, USA; 7 Zurich Neuroscience Center, Zurich, Switzerland; 8 Department of Neurological Surgery, The Ohio State University, Columbus, OH USA; 9 Department of Biomedical Engineering, The Ohio State University, Columbus, OH USA; 10 Institute of Precision Optical Engineering, School of Physics Science and Engineering, Tongji University, Shanghai, China; 11 Department of Precision Instrument, Tsinghua University, Beijing, China

**Keywords:** Neurovascular imaging, Two-photon microscopy, Fluorescence, Needle-beam

## Abstract

Comprehensive understanding of brain functions necessitates high-speed imaging of neuronal and vascular dynamics across extensive volumes. Functional neuroimaging investigations with two-photon microscopy are commonly hindered by its limited depth of field which restricts imaging rates across multiple planes. We introduce needle-shaped beam two-photon microscopy (NB-2PM), a versatile platform for high-throughput neurovascular imaging at sub-cellular resolution across multiple depths. It employs customized diffractive optical elements to generate single- or multi-plane needle beams with up to 10 times elongated depth of field relative to Rayleigh lengths and engineered axial energy distribution to effectively offset light attenuation with depth. The proposed method was applied to snapshot volumetric vascular imaging and multi-plane neurovascular dynamic recordings of resting state and stimulus-evoked activity in mice. NB-2PM can seamlessly be integrated into existing microscopy systems, thus providing a scalable platform for gaining comprehensive insights into the functional architecture of murine brain.

**Supplementary Information:**

The online version contains supplementary material available at 10.1186/s43074-026-00237-3.

## Introduction

Understanding brain functions requires dynamic tracking of multiple imaging targets, such as cerebral vasculature and neurons, which evolve into highly complex three-dimensional (3D) networks and operate collaboratively during brain activities. Dysfunctions in either neuronal or vascular networks are linked to a range of brain diseases, including stroke [1], Alzheimer’s [2, 3], and Parkinson’s disease [4]. Thus, there is a great need for a neuroimaging tool capable of delineating both the anatomical and functional organization of various compartments spanning a considerable depth range at cellular or subcellular levels.

Among a variety of neuroimaging tools, two-photon laser scanning fluorescence microscopy (2PM) [5, 6] has been extensively applied for preclinical brain research, attributed to its sub-micron resolution, multicolor imaging capability, and deep tissue penetration. Conventional 2PM systems typically utilized a high-numerical aperture (NA) objective to form a tightly focused Gaussian beam (GB) to enhance the nonlinear effect and optical sectioning. However, the high-NA excitation/detection regime restricts the depth of focus (DOF) to several microns. To capture fluorescence targets at multiple depths, axial scanning is required which can be achieved by shifting the imaging objective using a piezo stage, or by incorporating electrically tunable lenses [7–9] or tunable acoustic gradient-index lenses [10, 11] into the excitation light path. However, both approaches necessitate repetitive lateral scanning at each depth, limiting the temporal resolution for capturing 3D samples.

Spatio-temporal beam multiplexing represents another strategy to form axially separated foci with negligible switching time – typically several nanoseconds – by splitting the excitation beam into multiple beamlets passing through different excitation optical paths [12] or by integrating a reverberation optical loop [13]. The introduction of a partially transmissive mirror can further modulate the energy distribution among focal spots situated at different depths, thus compensating for light attenuation in deeper tissues [14]. The complexity of system design makes it challenging to integrate into other 2PM systems and the freedom to modulate the axial energy profile is also limited.

Another direction for rapid volumetric sampling replaces the conventional GB with a Bessel-like focus featuring an elongated DOF. This can be achieved using either an axicon [15] or a spatial light modulator (SLM) [16, 17] combined with an annular mask. The extended DOF facilitates simultaneous excitation of multiple fluorescent targets along the z axis, allowing a two-dimensional (2D) raster scan to render a projection view of the imaging volume without the need for additional axial scanning. However, the Bessel focus generated by axicons is subject to axial intensity oscillation due to fabrication imperfections at the axicon tip [18, 19]. In contrast, utilizing an SLM to display a concentric binary grating pattern guarantees full control over NA, DOF, and axial energy distribution [16, 17]. Despite these advantages, the bulky size and polarization-sensitive nature of SLM introduces complexities to the system design whilst the use of an annular apodization mask necessitates precise system alignment.

Here, we introduce a needle-shaped beam 2PM (NB-2PM) approach utilizing customized diffractive optical elements (DOEs) positioned directly in front of the objective lens. The considerable design flexibility of DOEs enables the generation of either single- or dual-plane NBs with an axially elongated focus, with tailored axial energy distribution to offset light attenuation in deeper tissues and balance the brightness variance across multiple fluorophores. We then demonstrate the application of single-plane NB-2PM for high-throughput structural and functional vascular imaging of the murine cortex, achieving near diffraction-limited resolution over a 60 µm depth range. Furthermore, we explore the potential of dual-plane NB-2PM for simultaneously monitoring the activity of both the pial network and neurons, axially displaced by 100 µm, during both resting state and whisker stimulation scenarios in awake mice. Owing to its simple design and high adaptability, the proposed method can seamlessly be integrated as an add-on module into most existing 2PM systems with matched beam size and NA to offer new insights into the interplay between neural activity and hemodynamic responses.

## Results

### NB-2PM design and system performance

**Fig. 1:**
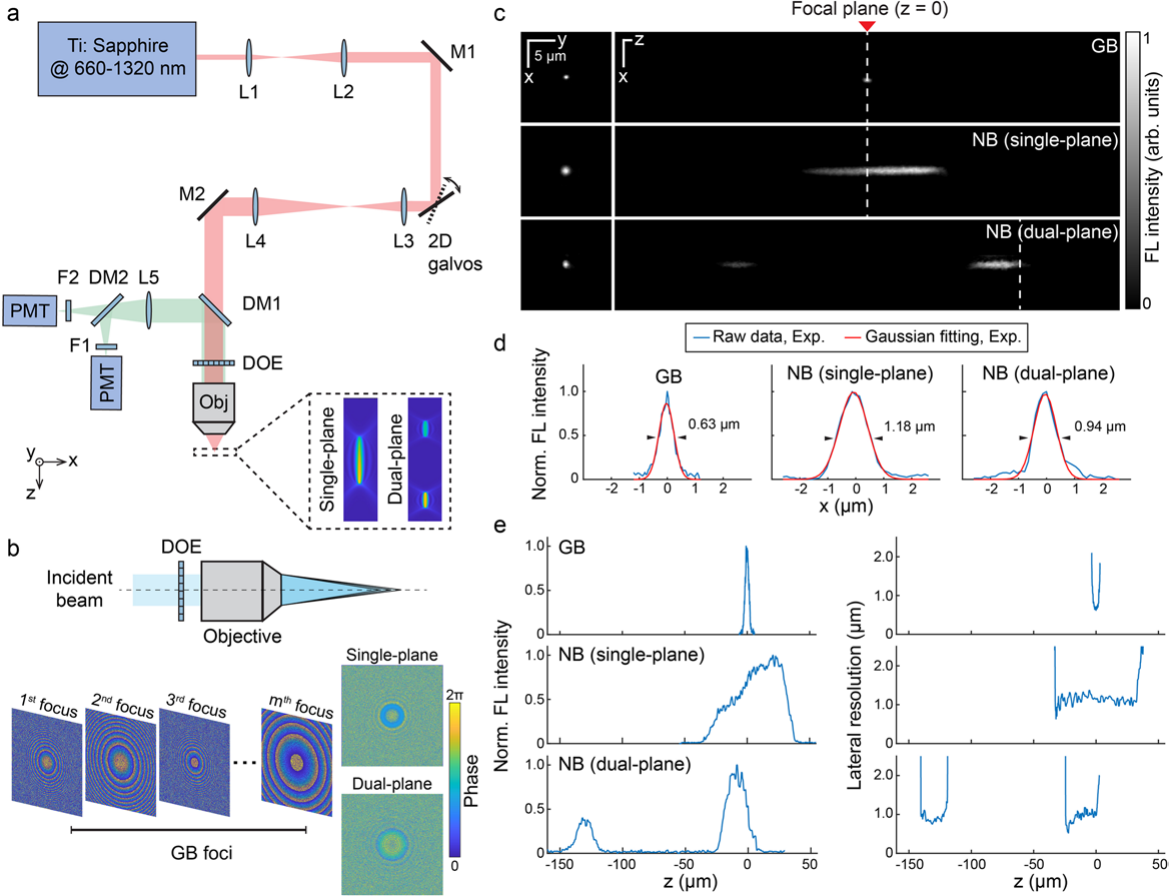
The needle-shaped beam two-photon microscopy (NB-2PM) system design and characterization. **a** Schematic of the NB-2PM setup. A customized DOE was positioned on top of the objective lens to generate either single-plane or dual-plane NB illumination. M, mirror; L, lens; DM, dichroic mirror; DOE, diffractive optical element; F, filter; PMT, photomultiplier tube; Obj, objective. **b** Design of DOEs and phase maps for single-plane and dual-plane NB configurations. The pixels on the DOE were grouped into multiple subsets, with each subset dedicated to generating a GB focus at a specific axial (z) location. The final phase map displayed on the DOE is rendered by summing up individual phase maps corresponding to each GB focus. **c** Lateral and axial PSFs measured by imaging 0.17 µm sized fluorescent beads under the GB versus NB illumination. The focal plane of the objective is designated as the zero reference point on the z axis. **d** Comparison of lateral resolution between the GB and NB cases. The lateral resolution was determined by the full-width-at-half-maximum of line profile in the lateral PSF following Gaussian fitting with MATLAB built-in “fit” function. **e** Comparative analysis of the fluorescence intensity distribution and lateral resolution as a function of z-axis position for the GB and NB configurations. 7.8 mW power was used for the GB configuration and 110 mW for the NB configuration

The simplified schematic of NB-2PM setup is depicted in Fig. 1a, where a customized DOE is integrated before the objective’s input in a standard 2PM system. This configuration produces an extended-focus needle-shaped beam (NB) within the imaged volume. Tailored for different imaging scenarios, the NB can either be distributed along a single plane (single-plane NB) or split into two separate planes (dual-plane NB). The underlying principle of DOE is to apply phase modulation to the input laser beam before it enters the objective, generating densely spaced GB foci along the z axis. This is achieved by dividing the DOE into multiple subsets of pixels, each dedicated to forming a single GB focus at a specific z position (Fig. 1b). We designed two specific DOEs for single-plane and dual-plane NB configurations, optimized for a central wavelength of 990 nm, with their phase patterns shown in Fig. 1b, bottom right. The number of GB foci is adjusted to 100 and 32 × 2 to achieve different beam lengths for single-plane and dual-plane cases, respectively. Theoretical simulations suggest that the single-plane NB spans a 60 µm depth range with 1.12 µm lateral resolution, while the dual-plane NB features two separate 20 µm long NBs with 0.87 µm lateral resolution, spaced 100 µm apart (Supplementary Fig. 1). The single-plane NB is centered around the original focus of the objective lens (f_0_), whereas the lower part of the dual-plane NB coincides with f_0_. The DOE design offers flexibility to position the NB at different axial positions relative to f_0_. Beyond the extended focus, we further modulate the axial energy distribution of NB by tuning the axial interval between every two adjacent GB foci (see online Methods for details), a feature crucial for in vivo imaging where tissue scattering can substantially degrade the signal-to-noise ratio (SNR). To maintain a comparable SNR across varying depths and fluorophores, the axial energy distribution of the NB was fine-tuned in both DOE designs. For the single-plane DOE, the axial energy distribution was designed under the assumption of exponential light attenuation in brain tissue, with a mean scattering-free path of ~ 150 µm [20]. Within a depth range of 60 μm, this exponential attenuation can be well approximated by a linear function. Accordingly, to compensate for depth-dependent attenuation, a linear energy gradient ranging from 0.67 to 1.0 was adopted to simplify the DOE design. For the dual-plane DOE, the energy distribution between the NB segments is set to a 0.4:0.6 ratio, accounting for the increased light attenuation in deeper tissues and varying fluorescence brightness of different fluorophores. While the attenuation properties and fluorescence brightness of fluorophores may vary under different imaging scenarios, the design flexibility of DOE allows customized axial profiles with tailored energy distributions.

We then characterized the system performance by imaging 0.17 µm sized fluorescent beads to quantify the lateral and axial point spread functions (PSFs) relative to GB illumination (Fig. 1c). The system design allows for easy switching between NB and GB modes through the addition or removal of the DOE. Higher input power was employed for the NB configurations to achieve a sufficient SNR for further analysis. The achieved lateral resolution was 1.18 µm for the single-plane NB and 0.94 µm for dual-plane NB, in contrast to 0.63 µm under the conventional GB mode (Fig. 1d). Moreover, the fluorescence intensity profile along the z axis displays a non-uniform distribution, as designed, with higher intensity in deeper regions (Fig. 1e, left). The DOF, determined as the depth range over which lateral resolution degrades by a factor of 2, has been significantly improved – 68.25 µm for single-plane and 24.57 µm for dual-plane NB, compared to 5.57 µm in the GB case (Fig. 1e, right). The experimentally measured system performance closely aligns with theoretical simulations, assuming that the two-photon fluorescence signal is proportional to the square of the illumination intensity (Supplementary Fig. 1).

### High-throughput structural and functional vascular imaging with attenuation correction over an extended focal range

**Fig. 2:**
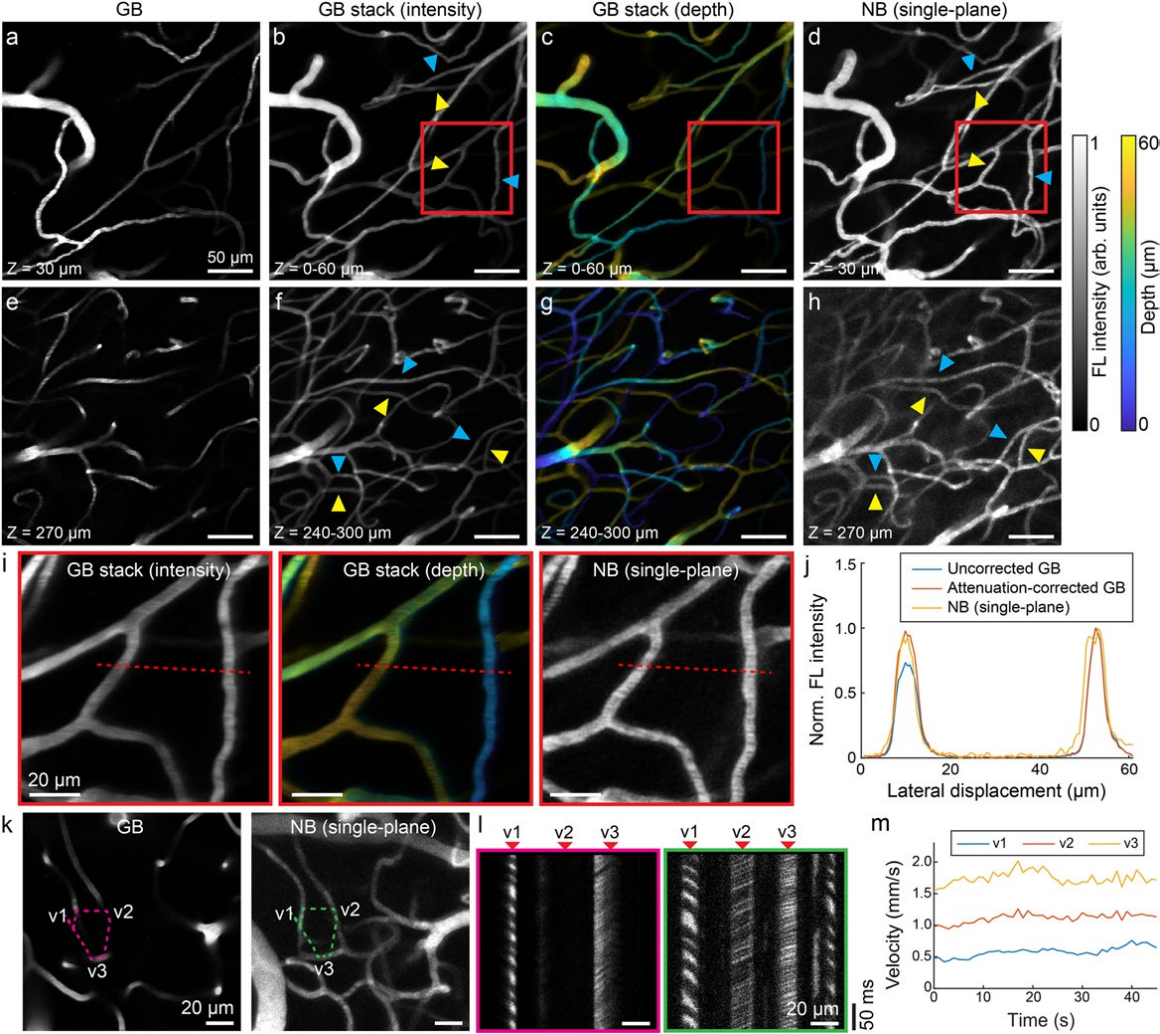
High-throughput structural and functional vascular imaging in murine brain with single-plane NB-2PM. **a**-**h** Microcirculation mapping of the murine brain following FITC-dextran injection using GB versus single-plane NB configurations. The data were collected at depth ranges of 0–60 µm and 240–300 µm. Images from left to right: GB single-slice image, sum of GB z-stack, color-encoded depth map derived from GB z-stack, NB single-slice image. Enhanced fluorescence intensity was observed in deep vessel branches (labeled with yellow triangles) compared to shallow vessel branches (labeled with blue triangles). **i** Zoom-in views of the region of interest (ROI) marked by a red square in (**b**-**d**). **j** Intensity line profile along the red dashed line in (**i**). **k** Vascular mapping in another brain region captured with the GB and NB configurations. An arbitrary scan path intersecting three vessels was chosen for line scanning, with corresponding kymographs shown in (**l**). **m** Temporal profiles of the velocity in three vessel branches obtained with NB-2PM. Representative data from one mouse are shown. 19 mW power was used for the GB configuration and 150 mW for the NB configuration

The extended focus of NB-2PM facilitates high-throughput imaging of realistic 3D tissue volumes, offering significant advantages over extensive axial scanning required by conventional 2PM. We then employed single-plane NB-2PM for vasculature mapping in mice implanted with cranial windows, following intravenous injection of FITC-dextran. For comparative analysis, the same brain region was imaged under both GB and NB configurations, whereas axial scanning of the objective lens was performed to render a z-stack image under GB mode. Structural comparisons were conducted at depth ranges of 0–60 µm and 240–300 µm below the brain surface (Fig. 2a-h). Owing to its limited DOF, the conventional GB image only captures a segmented view of the 3D vascular morphology (Fig. 2a). In contrast, NB-2PM enabled simultaneous visualization of a well-connected vascular tree spreading across a 60 µm depth range (Fig. 2d), showing vessel density comparable to a GB stack consisting of 30 image planes taken with 2 µm steps (Fig. 2b, c). The superiority of NB-2PM for cerebrovascular imaging is also observable at depths of 240–300 µm (Fig. 2e-h); however, the image contrast is partially reduced due to increased light attenuation in deep tissue and potential side lobe-induced excitation of out-of-plane vasculature arising from higher vascular density at greater depths. Due to intense light scattering in brain tissues, deeper vessels in the GB images exhibited lower fluorescence intensity compared to superficially located vessels of a similar diameter (labeled with yellow and blue triangles, respectively, in Fig. 2b, f). In contrast, NB-2PM images (Figs. 2d, h) manifested significant signal enhancement in deeper vessels, attributed to the engineered axial profile. To validate this, we applied digital attenuation correction to the GB z-stack by multiplying the image at each slice with a correction factor, which followed an exponential dependence with an attenuation length of 150 µm. Since fluorescence intensity of vessels correlates with their diameter under the extended-focus illumination [17], for the comparison we selected vessels with comparable diameters (4.84 and 4.96 μm) to mitigate the influence of diameter variability (Fig. 2i). The lateral profile of the NB-2PM image closely resembled the corrected GB data (Fig. 2j).

In addition to vessel morphology, we explored the potential of NB-2PM for functional vascular imaging when combined with arbitrary line scanning. The comprehensive view of vessel connectivity offered by NB-2PM facilitates simultaneous velocity measurements across multiple connected vessels, which could be selected by setting an arbitrary scan path (Fig. 2k). Subsequent line scanning along the predefined path allowed for directly comparing the kymographs recorded in the GB and NB modes, whereas dark streaks resulting from the negative contrast of flowing red blood cells were visible (Fig. 2l). Notably, all three vessels along the arbitrary line displayed high-contrast streaks in the NB-2PM image, with their temporally varying flow velocities calculated using Radon transform-based analysis (Fig. 2m). The maximal measurable flow velocity is theoretically determined by the system’s scanning speed and effective scanning length along the vessel centerline.

### Simultaneous, dual-plane neurovascular imaging in awake mice under resting state and whisker stimulation

**Fig. 3:**
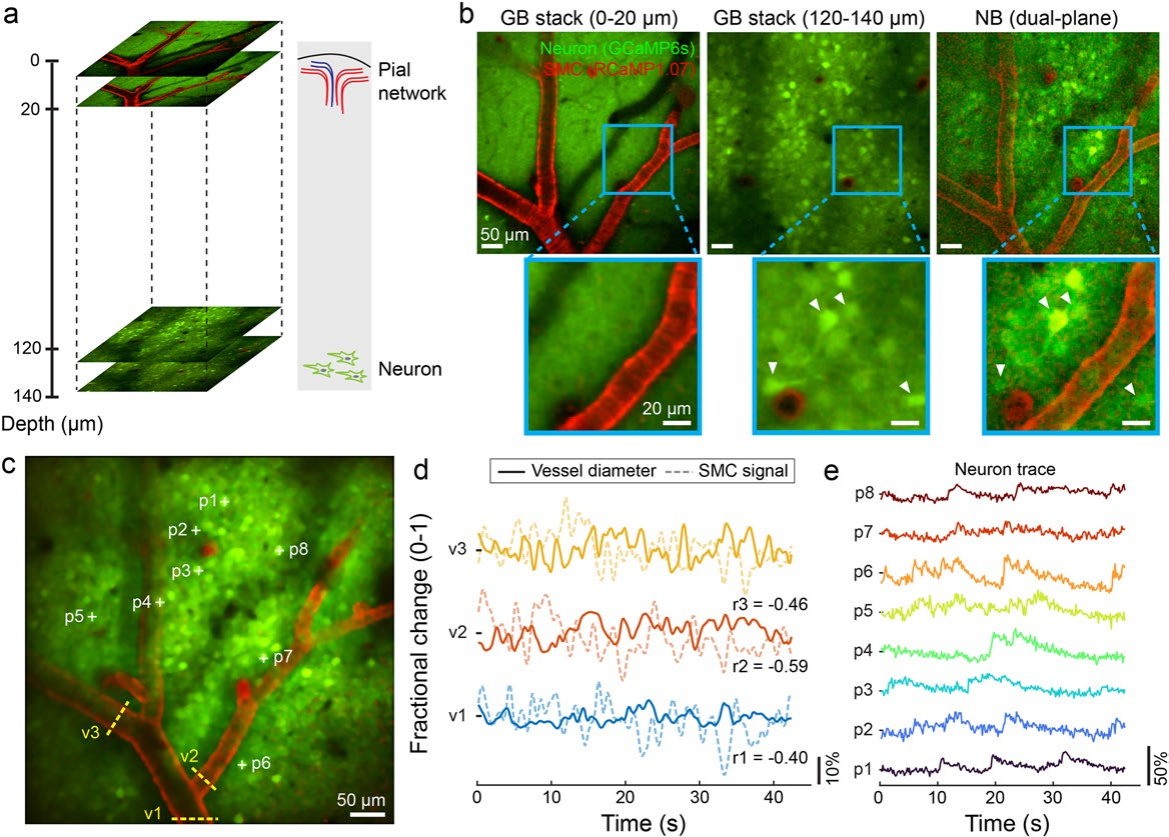
Simultaneous neurovascular imaging of resting state brain activity in awake mice captured with dual-plane NB-2PM. **a** Diagram of dual-plane NB-2PM for murine brain imaging, covering both superficial pial vasculature and deeper neuronal population with a 100 µm axial displacement. **b** Comparisons between GB and dual-plane NB images for a selected brain region. From left to right: sum of the GB z-stack at 0–20 µm depth range, sum of the GB z-stack at 120–140 µm depth range, NB-2PM image. **c** Structural map captured with NB-2PM for functional analysis. **d** Time courses of vessel diameter and SMC signal for three vessels (marked with dashed line in **c**), with Pearson’s correlation coefficients between the vessel diameter and SMC signal changes (r1 to r3) labeled. **e** Time courses of individual neurons marked with crosses in (**c**). Representative data from one mouse are shown. 13 mW power was used for the GB configuration and 150 mW for the NB configuration. For the GB illumination, the power at each depth was increased following an exponential power profile with a characteristic length constant (L_z_) of 120 ~ 150 μm

We next used the NB-2PM system to simultaneously monitor vascular and neuronal dynamics in the murine brain. During brain activation, responses commonly appear in both the superficial pial network and deeper neurons, underscoring the need for imaging techniques that can simultaneously track activities across different depth ranges. We thus implemented the dual-plane NB-2PM for simultaneous neurovascular imaging in awake head-fixed Acta2-RCaMP1.07 mice that express GCaMP6s in neurons and RCaMP1.07 in smooth muscle cells (SMCs) surrounding arterioles (Fig. 3a). In the GB case, z-stack images were sequentially collected at depth ranges of 0–20 µm and 120–140 µm with 2 µm steps (Fig. 3b, left and middle). As expected, GB images predominantly displayed pial arteries at shallower depths and a dense distribution of neurons in deeper layers. In contrast, NB-2PM enabled the visualization of structures across both depth ranges averting the need for axial scanning (Fig. 3b, right). In the NB image, both in-focus vessel structures and vessel shadows were observed: superficial pial vessels lying within the surface NB segment produce sharply defined dark regions, while deeper structures exhibited vessel shadows due to absorption by the overlying superficial vasculature (Fig. 3b). Similar vessel-related features were also observed in the corresponding GB images (left and middle, Fig. 3b). Functional readings of neurovascular dynamics were further performed with NB-2PM at a 6 Hz framerate (Fig. 3c), which was imperative for tracking changes in vessel diameter along with signal fluctuations in SMCs across selected vessel segments (Fig. 3d). The SMC signals inversely correlated with the vessel diameter change (Supplementary Fig. 2), as indicated by a negative Pearson’s correlation coefficient (r), consistent with the calculated r values observed in the GB case (Supplementary Fig. 3) and the literature [21, 22]. Moreover, spontaneous neuronal activations were captured in representative neurons (Fig. 3e). Structural comparison between GB and NB images (Supplementary Fig. 4a-c) revealed that the analyzed neurons were located within the 120 ~ 140 µm depth range. Furthermore, the vessel diameter measurements obtained with NB-2PM were consistent with those from GB images, although some variations were observed due to the elongated focus (Supplementary Fig. 4d).

**Fig. 4:**
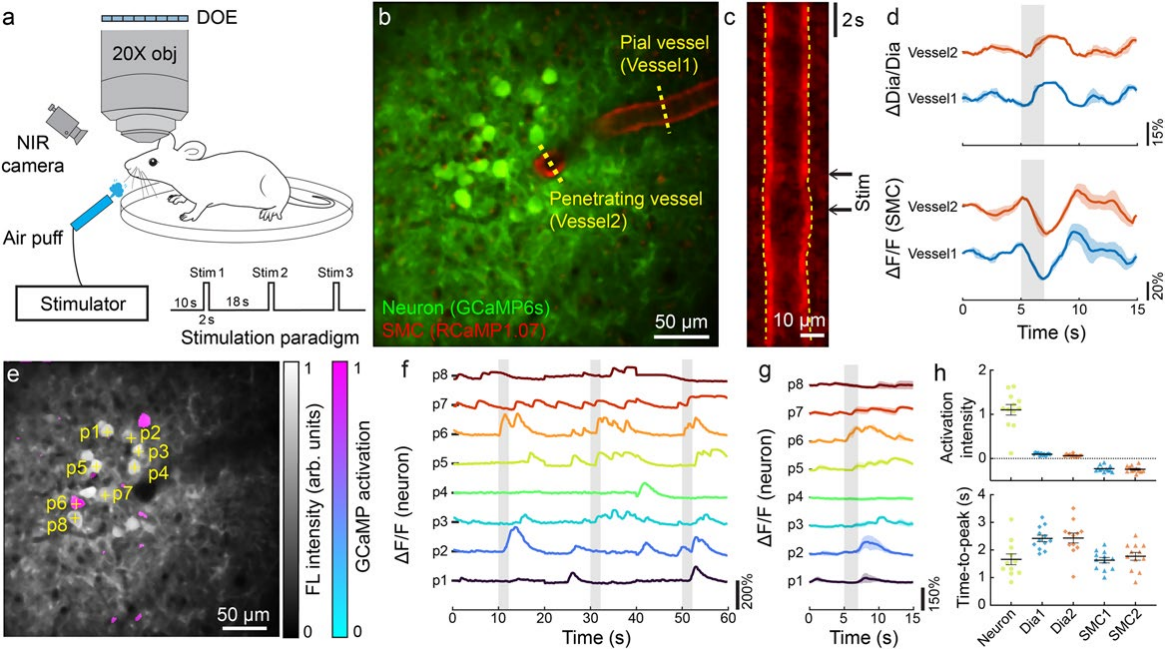
Neurovascular activation mapping during whisker stimulation in Acta2-RCaMP1.07 mice using dual-plane NB-2PM. **a** Diagram of dual-plane NB-2PM for capturing neurovascular activation in awake mice during air-puff whisker stimulation, following a predefined stimulation paradigm. **b** Structural map of the murine brain captured with NB-2PM. **c** Virtually rendered kymograph for the selected pial arteriole across a single stimulation cycle. **d** Trial-averaged vessel diameter and SMC activation curves of vessels labeled in (**b**). **e** GCaMP activation map overlaid on the structural map. The activation map was thresholded at 0.5 to delineate the activation region. **f** Time courses of individual neurons marked with crosses in (**e**). **g** Trial-averaged neuron activation curves. **h** Comparative analysis of activation intensity and response time of ROIs located on selected neuron and vessels. For vessel responses, both vessel diameter and SMC responses are included. Data are presented as mean ± s.e.m. Representative data from one mouse are shown. The experiment was repeated independently in three mice rendering similar results. 150 mW power was used for NB configuration

Next, we applied this method for neurovascular activation mapping in awake mice following whisker stimulation, employing air-puffs to unilaterally stimulate the whiskers according to a predefined paradigm (Fig. 4a, Supplementary Movie 1). During this process, the murine brain was imaged at a 6 Hz frame rate over a 60 s period, including 3 repetitive stimulation cycles. The structural map displayed both pial arteries and neurons, from which a pial arteriole and its connected penetrating vessel were selected for hemodynamic analysis (Fig. 4b). A virtual kymograph of the chosen pial arteriole was constructed by stacking line profiles of the vessel cross-section over time, revealing pronounced changes in vessel diameter following stimulation (Fig. 4c). These changes were further corroborated by a synchronized increase in vessel diameter and a decrease in SMC signals with the data averaged across *n* = 3 stimulation cycles (Fig. 4d). The vessel diameter changes were further validated with GB illumination (Supplementary Fig. 5). Furthermore, the neuron activation map was generated by calculating the Pearson’s correlation coefficient between neuron calcium signal changes and a boxcar stimulation pattern, which was superimposed onto the structural map (Fig. 4e). Representative neurons, located within the 120 ~ 140 µm depth range, were selected for further analysis (Supplementary Fig. 6). Traces from representative neurons are shown in Fig. 4f. Synchronized activation was observed in the 6th neuron in the averaged curves (Fig. 4g), consistent with the neuron activation map. The capability to concurrently monitor neuron and vessel dynamics at different depths allows for a detailed analysis of the spatio-temporal features of brain activation, by comparing the intensity and peak time of the neuron calcium signal, vessel diameter, and SMC signaling. Neurons exhibited higher activation intensity and shorter time-to-peak values compared to vessel diameter and SMC signaling (Fig. 4h), consistent with previous reports [23, 24]. We also applied NB-2PM in a Claudin5-eGFP mouse line, which manifested similar results (Supplementary Fig. 7).

## Discussion

In this study, we introduced NB-2PM featuring elongated DOFs with a compact and versatile design based on the integration of DOEs. Two configurations, namely single-plane and dual-plane NBs, are introduced with precise control over DOF, axial energy distribution, and axial displacement among NB segments. Different NB configurations can be tailored for specific imaging scenarios by considering the original 3D distribution of imaging targets. Initially, we applied the single-plane NB-2PM for high-throughput vascular imaging in the murine brain, capturing fully connected vessel networks across multiple depths in a single snapshot. This capability further facilitates flow velocity measurements across adjacent vessel branches via an arbitrary line scanning method. A dual-plane configuration was then employed for more complex imaging scenarios involving multiple imaging targets at distinct depths. We showcased simultaneous dynamic observations of both superficial pial network and deeper neuronal populations in awake mice, delivering real-time readings of vessel diameter change, SMC signaling, and neuronal activity.

Compared to previously reported Bessel-beam 2PM systems utilizing axicons [18] or SLMs [16, 17], DOEs offer a simpler and more cost-effective solution for generating axially extended foci with minimal modifications to the original 2PM system. The implementation of NB-2PM requires only the insertion of a fabricated DOE, which can be manufactured in a lithography facility at an approximate cost of ~ 100 USD, without the need for additional optical components. Although NB-2PM exhibits relatively lower theoretical lateral resolution, it benefits from a suppressed side lobe ratio (defined as the first side lobe to the central lobe) compared to reported Bessel-beam 2PM systems [16], resulting in reduced fluorescence background. Multi-plane imaging has also been demonstrated in Bessel-beam 2PM by incorporating a remote-focusing module [25]; however, this approach does not allow for simultaneous multi-plane acquisition. Moreover, the addition of a remote-focusing module increases system complexity and introduces additional energy loss. Comparatively, the dual-plane DOE achieves simultaneous multi-plane imaging with a single compact component. Another method to generate the NB involves reducing the system’s NA by illuminating only sub-apertures of the back pupil of the imaging objective [26]. Scanning different sub-apertures allows for the acquisition of multiple angular projections of the sample and retrieval of 3D volumes through tomographic reconstruction. However, the increased complexity of optical design and alignment poses significant challenges for integrating this approach into existing 2PM systems.

Compared with multifocal and needle-shaped beam approaches previously employed for OCT studies [27, 28], our NB-2PM approach features several important distinctions. Through tailored DOE design, we achieved flexible axial profiles either by adjusting the spacing between adjacent GB foci (single-plane) or allocating separate DOE pixel groups to different imaging planes (dual-plane). While NB-OCT employed DOEs designed to produce uniform axial profiles [27], in the single-plane NB-2PM method the axial profile is tailored to a linear distribution by iteratively optimizing the spacing between adjacent GB foci, thus amplifying signals from deeper layers. The multifocal OCT approach [28] used instead metasurfaces to generate multiple separated GB foci, whereas our dual-plane NB-2PM creates two separated NB segments instead of two GB foci, which is specifically designed to overcome the limited DOF of conventional 2PM. In addition, employing NB for 2PM inherently suppressed side lobe ratios owing to the nonlinear excitation process. Importantly, NB-2PM has also broadened the range of biological applications, enabling structural and functional imaging of vascular and neuronal systems, including simultaneous flow measurements and real-time assessment of neurovascular coupling in vivo.

The lateral resolution of NB-2PM could be tuned by utilizing different DOE designs. Simulations indicate that the optimal achievable lateral resolution with the NB DOE is 0.76 µm, approaching the diffraction limit of the GB case. However, the energy efficiency of DOE (energy in the main lobe) tends to decrease as lateral resolution increases. The two DOEs used in this work feature the lateral resolution of ~ 1 μm, selected to resolve capillary and neuronal structures in our application scenarios. The extended focus of NB-2PM could result in potential signal overlap along the z axis, especially under conditions of dense fluorescent labeling. In our quantification using transgenic GCaMP mouse brains, the overlap ratio for neuronal imaging ranged from 0–14%, depending on the neuron density in specific depths (Supplementary Fig. 8). This limitation can be alleviated by sparse-labeling strategies, such as labeling only a subpopulation of neurons, which could substantially reduce overlap and thereby improve NB-2PM performance (Supplementary Fig. 7), particularly when employing a longer DOF.

Several technical advancements are envisioned to enhance the imaging performance of NB-2PM. Currently, the energy efficiency of DOEs for NB-2PM is relatively low, limiting the maximum imaging depth to ~ 300 µm with the available laser power. To improve energy efficiency, one potential approach is to further reduce the pixel size of fabricated DOEs, thereby increasing the freedom to modulate the input beam. The elongated DOF feature of NB-2PM, whilst advantageous for high-throughput imaging, inevitably leads to reduced axial resolution. The missing depth information could be compensated by acquiring a z-stack image of the same sample under GB excitation. Another potential solution is to utilize multi-view excitation to reconstruct a more comprehensive 3D view [26, 29, 30]. Even though the light attenuation is compensated by employing an exponential-distributed axial energy deposition in the NB design, wavefront distortions caused by inhomogeneities in refractive index may induce PSF distortions. The latter could potentially be mitigated by implementing adaptive optics to maintain diffraction-limited performance in scattering samples [31], albeit at the expense of increased system complexity. Concerning temporal resolution, the imaging speed of NB-2PM is ultimately determined by the lateral scanning speed. Acceleration can be achieved via resonant galvo or polygon scanners [32], as well as multifocal illumination [33] or temporal beam multiplexing [34, 35].

## Conclusion

We introduced NB-2PM method incorporating customized DOEs as an add-on module with two available configurations. The single-plane NB-2PM was applied for both structural and functional vascular imaging with extended focus capabilities. Furthermore, the dual-plane configuration was utilized to concurrently monitor neuronal and vascular activities in awake mice under resting state and whisker stimulation conditions. The new approach offers a wealth of possibilities for advanced neurovascular studies in the murine brain.

## Methods

### NB-2PM setup

The NB-2PM system diagram is depicted in Fig. 1a, which was built on a custom 2PM system described elsewhere [36]. The system utilized a Ti:Sapphire optical parametric oscillator (OPO) laser (Chameleon Discovery NX, 660–1320 nm, Coherent, USA), featuring ~ 100 fs pulse duration and 80 MHz repetition rate for two-photon excitation. An XY galvanometer scanner (86-809-6215H, Cambridge Technology, USA) was used for beam steering. A combination of a scan lens (AC508-075-B, Thorlabs, USA) and tube lens (AC508-300-B and AC508-400-B, Thorlabs, USA) conjugates the galvos to the back focal plane (BFP) of a 20 × water-immersion objective (W-Plan Apochromat 20 ×/1.0 DIC, NA 1.0, back aperture 16.5 mm, Carl Zeiss, Germany), mounted on a piezo motor-driven linear stage (P-628.1CD, Physik Instrumente, Germany). The objective was underfilled with a beam size of 12 mm, corresponding to an effective NA of 0.73. The system supports two illumination configurations on the sample plane – single-plane NB and dual-plane NB – selectable by switching the customized DOE positioned at the back aperture of the objective casing. The backscattered fluorescence was collected by the same objective and passed through the DOE. A dichroic mirror (FF520-Di02-50 × 50, Semrock, USA) was used to separate the excitation and fluorescence detection light path. The deflected fluorescence was then focused by an aspheric condenser lens (ACL5040-A, Thorlabs, USA) and filtered by a cascade of a bandpass filter (FF01-535/50 or FF01-607/70, Semrock, USA) and shortpass filter (HC 770/SP, Semrock, USA) before detection by a photomultiplier tube (PMT, H10770B-40/-50, Hamamatsu, Japan). The PMT signal was sampled with a high-speed digitizer (PCI-6229, National Instruments, USA) and transferred to a PC. Data acquisition was performed with ScanImage software platform [37] (r3.8.1, Janelia Research Campus, USA).

### Design and fabrication of DOEs

The general idea of forming a NB consists in generating densely spaced GB foci along the axial direction [27, 38]. The step-by-step workflow for DOE design is detailed in Supplementary Note 1. Following the Abbe sin condition [39], the phase profile of the objective ${P}_{obj}(x,y,f)$ can be represented via

1 $$P_{obj}(x,y,f)=\frac{2n\pi}\lambda(\sqrt{f^2-\left(x^2+y^2\right)}-f),$$

where $(x,y)$ denotes the lateral coordinates, $f$ stands for the focal position in the medium, n is the refractive index of the medium, $\lambda$ is wavelength. To shift the focus from its original location ${f}_{0}$ to ${f}_{m}$ (the focal position of ${m}^{th}$ GB focus), the phase pattern at the BFP is designed as follows.

2 $${P}_{DOE}= \sum\nolimits_{m=1}^{M}({P}_{obj}(x,y,{f}_{m})-{P}_{obj}(x,y,{f}_{0})-{P}_{{a}_{m}})\times {L}_{m}(x,y)$$

Here, M represents the total number of foci, m is the index of focus, ${P}_{{a}_{m}}$ is the phase regulator responsible for beam diameter modulation, ${L}_{m}(x,y)$ is a binary matrix indicating the pixels allocated to form the ${m}^{th}$ focus. The 1 values in ${L}_{m}(x,y)$ are distributed randomly to suppress high-order diffraction noise, ensuring low background and high SNR. The number of 1 values determines the amount of input light energy allocated to the ${m}^{th}$ focus. The length and axial location of a NB are controlled by ${f}_{M}-{f}_{1}$, with an average interval between two adjacent foci empirically chosen to be around 0.8 times Rayleigh length of the objective for a smooth axial intensity profile. The DOE contains 1024 × 1024 pixels in total, each with a 10 µm pixel size.

By optimizing axial locations of the foci (${f}_{1}$, …,${f}_{M}$), the axial energy distribution of an NB or NB segment can be tuned. With fixed locations for the first and last foci, an iterative algorithm was developed to adjust individual axial interval between all the pairs of adjacent foci. Modifying the interval can decrease or enhance the local light intensity, achieving either a linear or uniform distribution in the single-plane and dual-plane DOE designs studied in this work. In addition, the second DOE comprises two segments, with 45% and 55% of pixels allocated to the first (shallow) and the second (deep) NB segments respectively, yielding an energy ratio of (45/55)^2^ = 2:3. The efficiency of DOE, quantified as the ratio of energy deposited within the main lobe of the NB relative to the input GB, is 5.65% and 11.04% in the single-plane and dual-plane NB cases, respectively. Additionally, the side lobe ratio is 12% for the single-plane NB and 8% for the dual-plane NB, as determined from beam profile simulations.

The DOE was fabricated with a four-round lithography (1100 nm, 550 nm, 275 nm, and 138 nm corresponding to π, π/2, π/4, and π/8, respectively) on a fused silica wafer, to create the designed phase pattern. The phase modulation depends on the structure height $H(x,y)$ and refractive index difference between air (${n}_{air}$) and fused silica (${n}_{silica}$), formulated as $2\pi H\left(x,y\right)\times ({n}_{silica}-{n}_{air})/\lambda$. The fabrication was carried out at the Stanford Nanofabrication Facility, with detailed fabrication process listed in Supplementary Note 2.

### System characterization

To characterize the lateral resolution and DOF of the system, fluorescence beads with ~ 0.17 µm diameter (505/515 nm, F8811, ThermoFisher, USA) were dispersed on a microscope slide. The beads were then imaged under both GB and NB illumination. A z-stack image was collected by translating the objective lens along the z axis with a step size of 2 µm.

### Animals

#### Animal models

All animal experiments were performed in accordance with the Swiss Federal Act on Animal Protection and approved by the Cantonal Veterinary Office Zurich. For vessel imaging, *n* = 3 C57BL/6 mice (8–40 weeks old, mixed sexes) were used. For experiments involving awake imaging under resting state and during whisker stimulation, *n* = 3 Acta2-RCaMP1.07 (Jackson Strain #:028345) mice (8–40 weeks old, mixed sexes) and *n* = 1 Claudin5-eGFP mouse (12–30 weeks old, female) were imaged. For quantification of the overlap ratio between structures in the superficial and deep planes, *n* = 1 Rasgrf2-2A-dCre; Ai148D mouse (36 weeks old, male) was imaged with the dual-plane configuration. This double transgenic strain expresses GCaMP6f in layer 2/3 cortical neurons. The mice were housed in ventilated cages with ad libitum access to food and water. The housing facility maintained a 12 h dark/light cycle, 22 °C room temperature, and ~ 50% relative humidity.

#### Chronic cranial window implementation and adeno-associated virus (AAV) injection

Mice were anesthetized intraperitoneally with a mixture of fentanyl (0.05 mg/kg, Sintenyl, Sintetica), midazolam (5 mg/kg; Dormicum, Roche), and medetomidine (0.5 mg/kg, Domitor, Orion Pharma). During the surgical process, the mouse head was fixed in a stereotaxic frame with its body temperature maintained at ~ 37 °C using a feedback-regulated heating system. The scalp was gently removed, followed by 4 × 4 mm craniotomy above the primary somatosensory cortex using a dental drill (Bien-Air). 300 nL of AAV vector (pssAAV-2-hSyn1-chI-GCaMP6f-WPRE-SV40p(A) 4.4 × 10^12^ VG/ml, Viral Vector Core Facility, University of Zurich) was then injected into the cortex tissue at a depth of ~ 300 µm. A square coverslip along with a titanium head plate was subsequently fixed to the skull with dental cement. The mice recovered for 7 days before being trained to awake head fixation.

#### Preparation for imaging session

For vessel imaging, mice were anesthetized with isoflurane (3–4% for induction, 1.5% for maintenance) in a mixture of oxygen and medical air with flow rates of 0.4 L/min and 0.6 L/min, respectively. Prior to the imaging session, 50 µL 2.5% FITC dextran (90718, Sigma Aldrich, USA) was intravenously injected for vascular imaging.

#### Whisker stimulation

Whiskers on the left side were stimulated using air puff (Picospritzer III, Parker, USA), which were controlled by a trigger box (STG4000 Series, MCS Stimulus Generators, Warner Instruments LLC, USA). During the imaging session, the mouse was positioned on a plate and head-fixed under the objective to mitigate motion artifacts. Each stimulation cycle consisted of 10 s baseline, 2 to 4 s stimulation, and 8 s post-stimulation period. Each imaging session included 3 repetitive stimulation cycles. A near-infrared (NIR) camera was used to monitor and record the animals’ behavior during data acquisition. The recorded videos were inspected to identify excessive body motion and self-generated whisker grooming, which could confound neurovascular responses evoked by externally delivered whisker stimulation and thereby affect the analysis.

### Data analysis and statistics

#### Arbitrary line scan analysis

For the analysis of arbitrary line scan data, the kymograph for each vessel was first cropped from the original image. Only kymographs showing visible dark streaks were selected for further analysis. The flow velocity was then quantified using an open-source CHIPS toolbox with Radon-transform based method [40].

#### Vessel diameter measurement

The vessel diameter measurements in Fig. 2 were conducted with the “vessel diameter.ijm” plugin (version 1.0) in ImageJ (version 1.54d, National Institutes of Health, USA) [41]. To measure the vessel diameter in Figs. 3 and 4, we plotted line profiles along the vessel cross-sections, which displayed two distinct peaks near the vessel walls. A Gaussian fitting was applied to identify the precise peak positions. The distance between these two peaks was defined as the vessel diameter in this study, representing the outer vessel diameter.

#### GCaMP activation map calculation

The acquired image stack was initially registered to eliminate motion artifacts. The image co-registration was conducted using the built-in “imregcorr” function in MATLAB (R2020b, Mathworks, Natick, MA, USA), relying on structural information derived from the vessel channel. Following image co-registration, the GCaMP activation map was computed by calculating the Pearson’s correlation coefficient between the GCaMP time courses and the predefined boxcar stimulation pattern, on a pixel-by-pixel basis. The generated GCaMP activation map was thresholded at a value of 0.5 before being fused with the structural map for better visualization.

## Supplementary Information

Supplementary Material 1.

Supplementary Material 2: Supplementary Movie 1. Neurovascular activation mapping during whisker stimulation in Acta2-RCaMP1.07 mice with NB-2PM.

## Data Availability

The main data supporting the finding of this study are available within the main text or supplementary information. The raw NB-2PM datasets are available for research purposes from the corresponding author upon request. Data analysis was performed with custom MATLAB codes which are available for research purposes from the corresponding author upon request.

## Abbreviations

NB-2PM: Needle-shaped Beam Two-Photon Microscopy
3D: Three-Dimensional
2PM: Two-Photon Microscopy
NA: Numerical Aperture
GB: Gaussian Beam
DOF: Depth of Focus
SLM: Spatial Light Modulator
2D: Two-Dimensional
DOE: Diffractive Optical Element
NB: Needle-shaped Beam
SNR: Signal-to-Noise Ratio
PSF: Point Spread Function
SMC: Smooth Muscle Cell
OPO: Optical Parametric Oscillator
BFP: Back Focal Plane
PMT: Photomultiplier Tube
AAV: Adeno-Associated Virus
NIR: Near-infrared
ROI: Region of Interest
