# Supplementary material for: Needle beam two-photon microscopy for simultaneous multiplane neural and vascular imaging in awake mice
Source: Photonix. 2026 Mar 16;7(1):18. doi: 10.1186/s43074-026-00237-3 (PMC12992400; doi:10.1186/s43074-026-00237-3)
Supplement: Supplementary file 1 — Supplementary Material 1. [file 43074_2026_237_MOESM1_ESM.docx]

Supplementary Information for

**Needle beam two-photon microscopy for simultaneous multiplane neural and vascular imaging in awake mice**

Quanyu Zhou^1,2,†^, Jingjing Zhao^3,4,†^, Chaim Glück^1,7,†^, Yu-Hang Liu^1,2^, Lin Du^8,9^, Lukas Glandorf^1,2^, Tian Jin^1,2^, Zhenyue Chen^10^, Lingqi Jiang^11^, Bruno Weber^1,7,*^, Adam de la Zerda^5,6,*^, Daniel Razansky^1,2,7,*^

^1^ Institute of Pharmacology and Toxicology, Faculty of Medicine, University of Zurich, Zurich, Switzerland

^2^ Institute for Biomedical Engineering, Department of Information Technology and Electrical Engineering, ETH Zurich, Zurich, Switzerland

^3^ School of Medical Equipment Science and Engineering, Huazhong University of Science and Technology, Wuhan, 430074, China

^4^ College of Life Science and Technology, Huazhong University of Science and Technology, Wuhan, 430074, China

^5^ Department of Structural Biology, Stanford University School of Medicine, Stanford University, Stanford, USA

^6^ Biophysics Program, Molecular Imaging Program, and Bio-X Program at Stanford University, Stanford, USA

^7^ Zurich Neuroscience Center, Zurich, Switzerland

^8^ Department of Neurological Surgery, The Ohio State University, Columbus, OH, 43210, USA

^9^ Department of Biomedical Engineering, The Ohio State University, Columbus, OH, 43210, USA

^10^ Institute of Precision Optical Engineering, School of Physics Science and Engineering, Tongji University, Shanghai, China

^11^ Department of Precision Instrument, Tsinghua University, Beijing, China

^†^ These authors contributed equally

^*^ Correspondence: daniel.razansky@uzh.ch, adlz@stanford.edu, bweber@pharma.uzh.ch


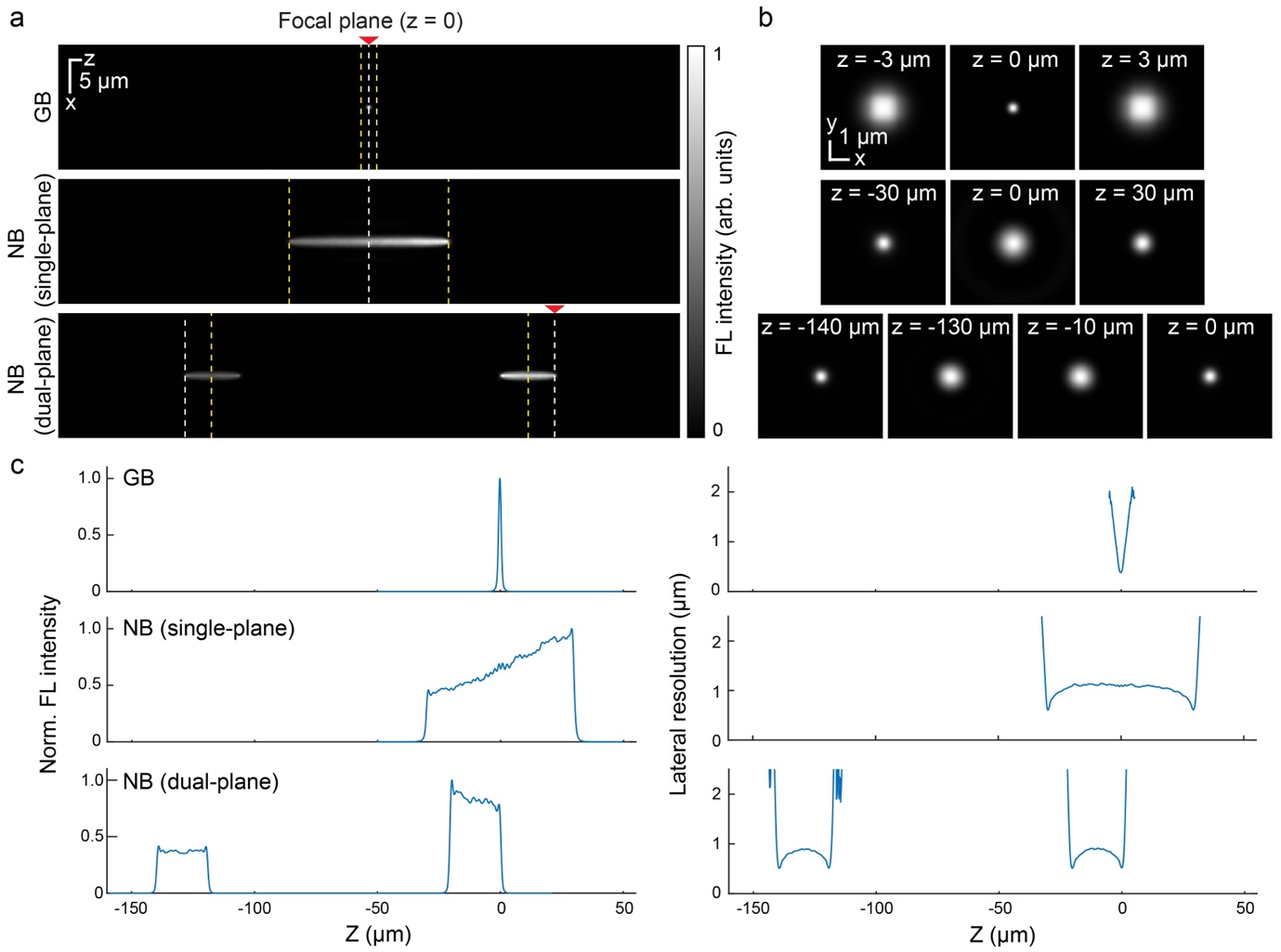


**Supplementary Figure 1. Simulated lateral and axial point spread functions (PSFs). (a)** Simulated axial PSFs under GB and two NB configurations, assuming a linear relationship between two-photon fluorescence signals and the square of excitation beam intensity, with lateral PSFs at different depths shown in **b**. **(c)** Comparative analysis of the fluorescence intensity distribution and lateral resolution as a function of z-axis displacement for GB and NB configurations.


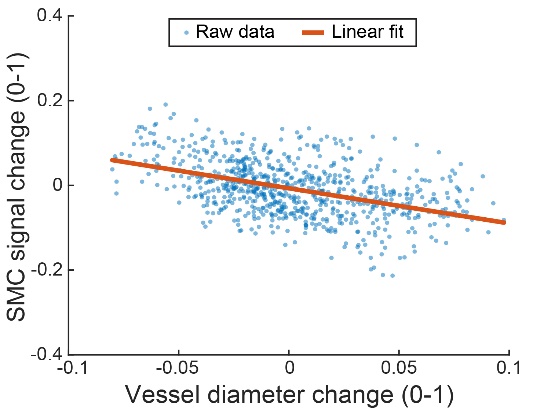


**Supplementary Figure 2. Scatter plot between vessel diameter and SMC signal change recorded with NB-2PM.**


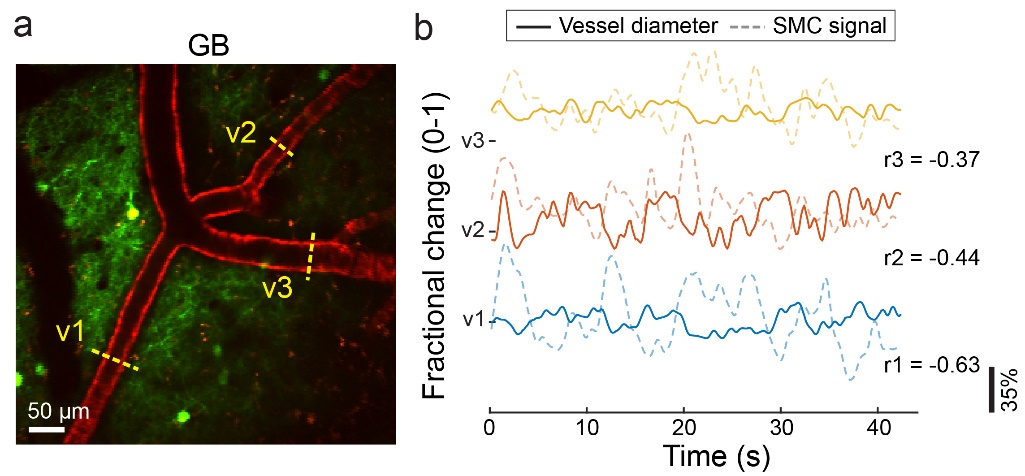


**Supplementary Figure 3. Brain imaging of resting-state vascular activity in an awake Acta2-RCaMP1.07 mouse with GB illumination.** **(a)** Structural map obtained with GB illumination. **(b)** Time courses of vessel diameter and SMC signal change for three vessels (marked with dashed line in **a**), with Pearson’s correlation coefficients (r1 to r3) between the vessel diameter and SMC signal change labeled.


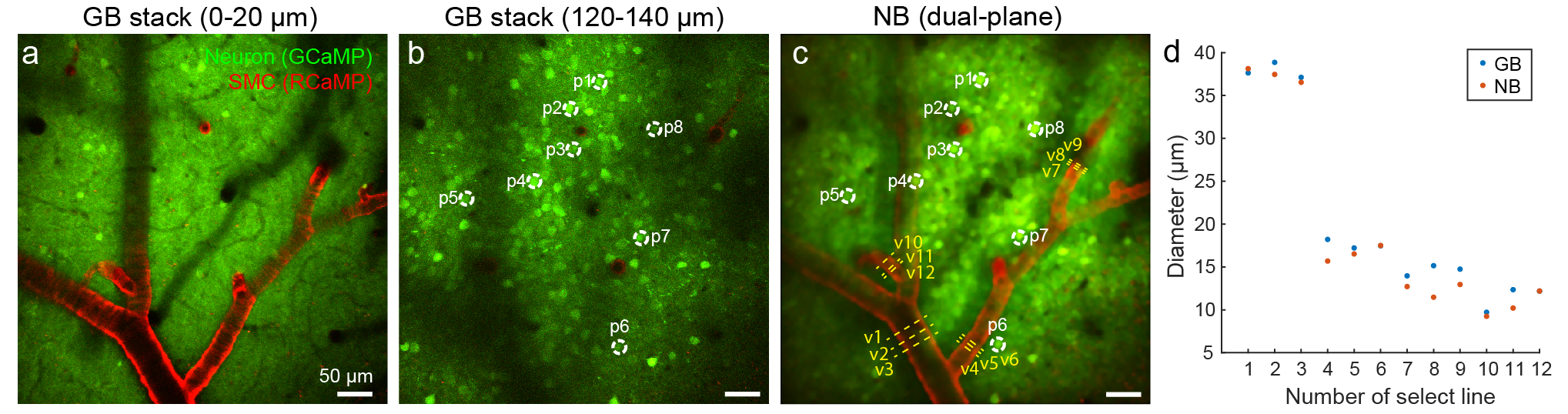


**Supplementary Figure 4.** **Comparison between GB and dual-plane NB images in an Acta2-RCaMP1.07 mouse under resting state. (a, b)** sum of the GB z-stack images from depth ranges of 0-20 µm and 120-140 µm, respectively. **(c)** Dual-plane NB image captured at a depth of 140 µm. The same neurons analyzed in Fig. 3e are labeled with circles in **b** and **c**. **(d)** Vessel diameter comparison between a GB single-slice image (at z = 10 μm) and NB image along the selected lines indicated in **c**.


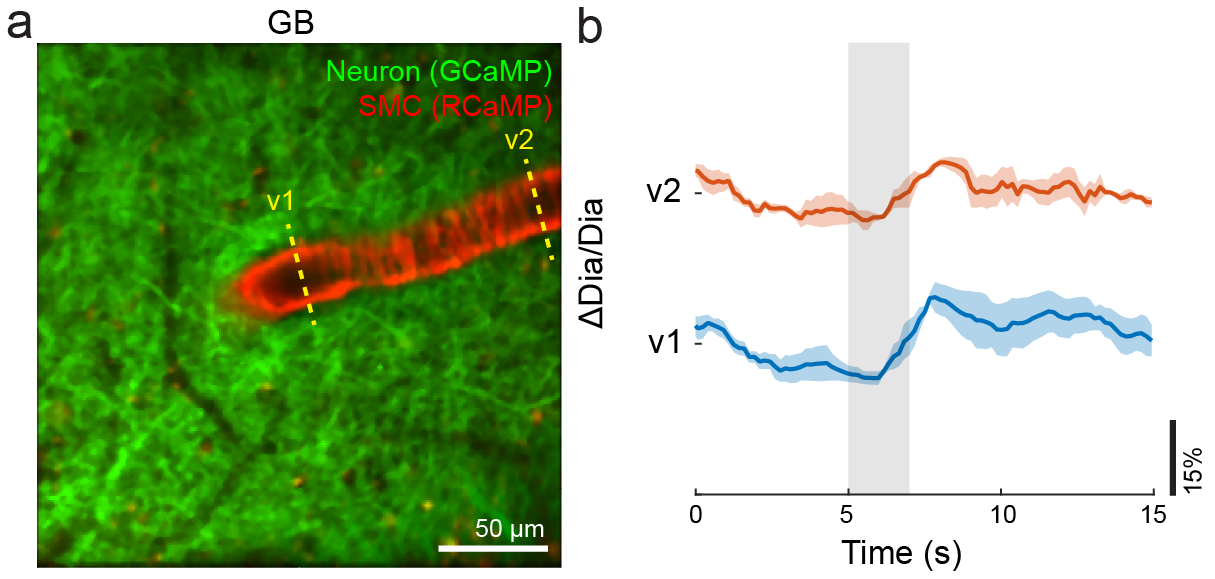


**Supplementary Figure 5.** Cerebrovascular imaging in an Acta2-RCaMP1.07 mouse post whisker stimulation with GB illumination. **(a)** Structural map of the murine brain captured with GB illumination. **(b)** Trial-averaged diameter change of the vessels labeled in **a**.


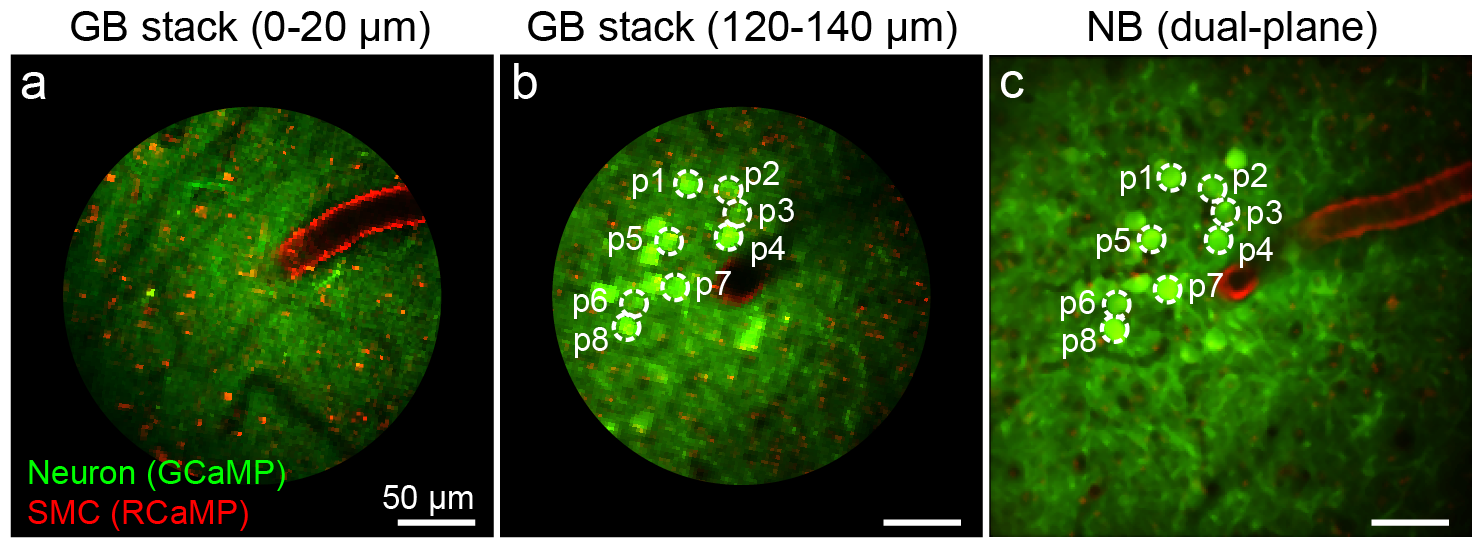


**Supplementary Figure 6.** **Comparison between GB and dual-plane NB images in an Acta2-RCaMP1.07 mouse under whisker stimulation. (a, b)** sum of the GB z-stacks from depth ranges of 0-20 µm and 120-140 µm, respectively. **(c)** Dual-plane NB image captured at a depth of 140 µm. The same neurons analyzed in Fig. 4e are labeled with circles in **b** and **c**.


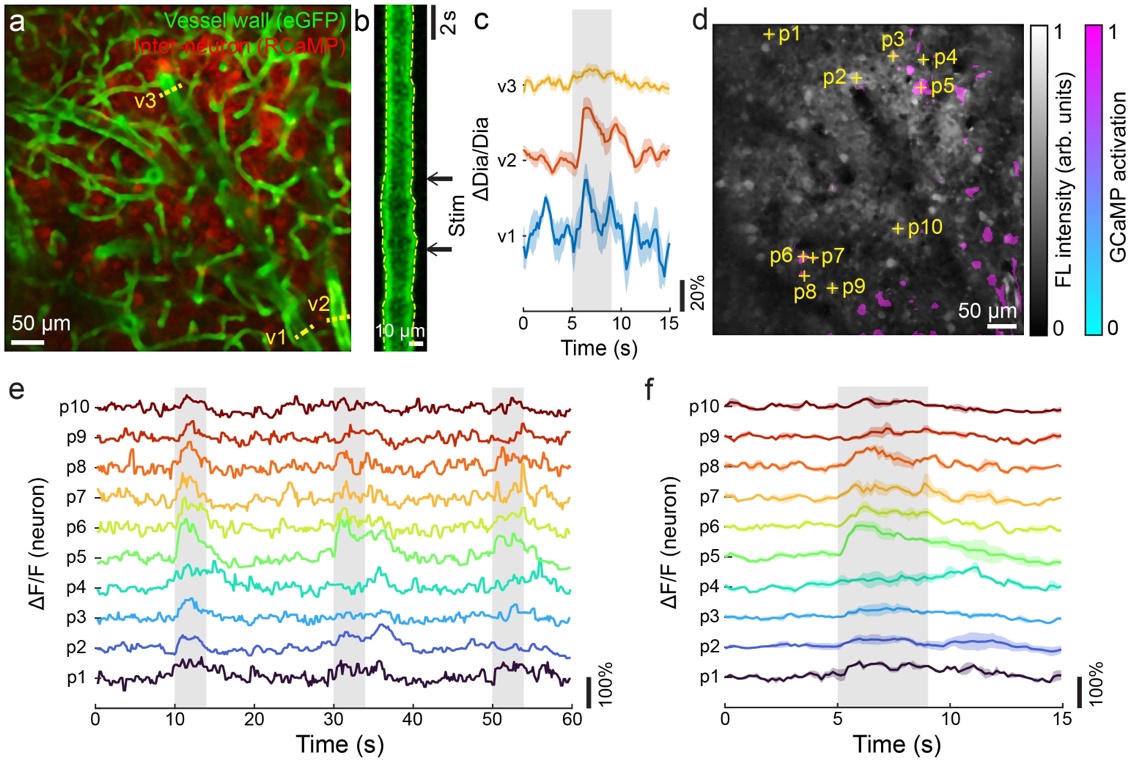


**Supplementary Figure 7. Neurovascular activation mapping during whisker stimulation in a Claudin5-eGFP mouse with dual-plane NB-2PM. (a)** Structural map of the murine brain captured with NB-2PM. **(b)** Virtually rendered kymograph for the selected pial vessel across a single stimulation cycle. **(c)** Trial-averaged vessel diameter activation curves of vessels labeled in **a**. **(d)** Neuronal activation map overlaid on the structural map. **(e)** Time courses of individual neurons marked with crosses in **d**, with trial-averaged curves shown in **f**. Data are presented as the mean ± s.e.m. Representative data from one mouse are shown.


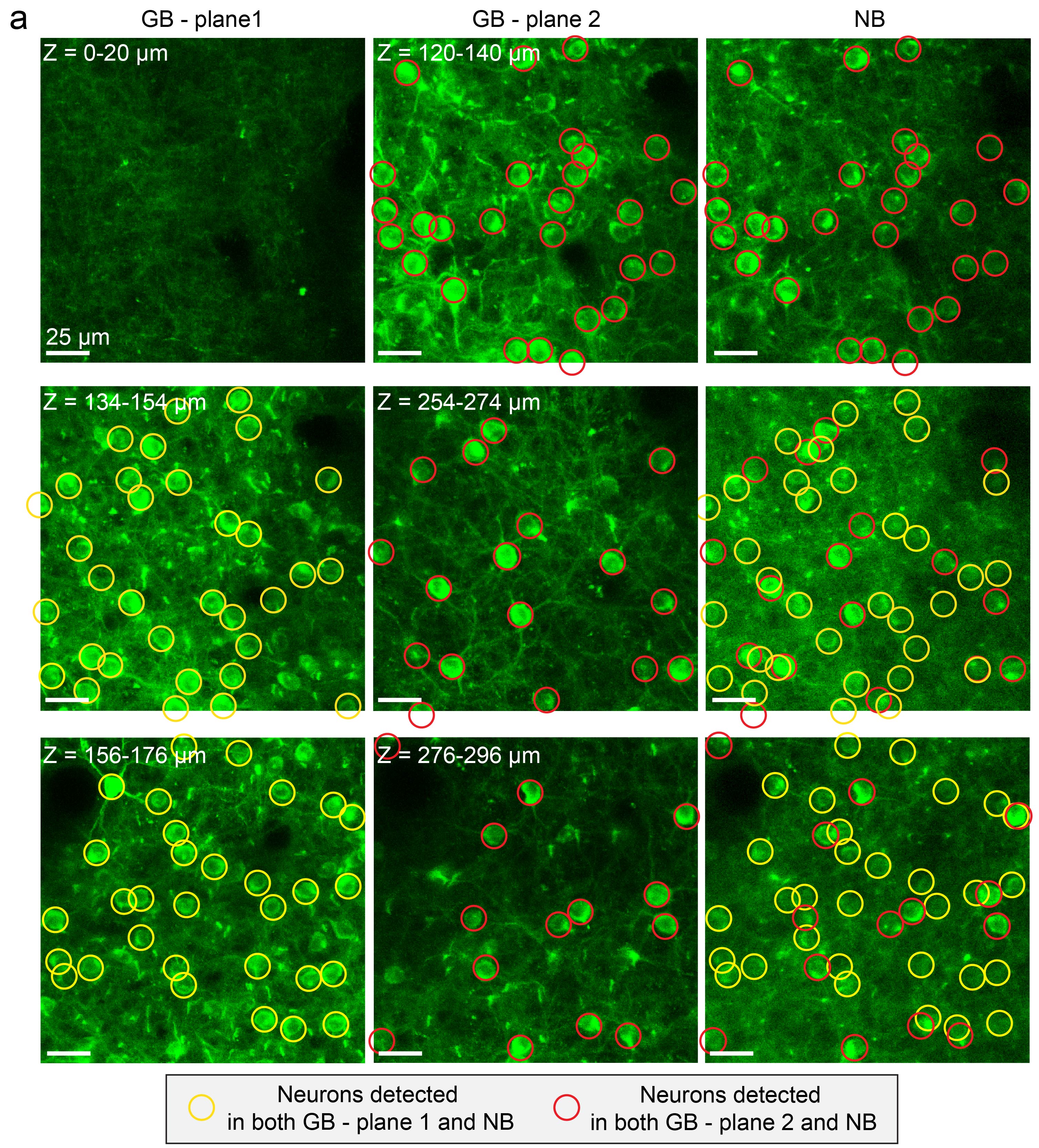


**Supplementary Figure 8. Overlap between superficial plane (plane 1) and deep plane (plane 2) when comparing the GB and NB images, obtained by imaging the brain of a** **Rasgrf2-2A-dCre; Ai148D mouse at different depths. Neuron somata detected in both GB (plane 1 or 2) and NB images are indicated with circles.**

**Supplementary Note 1. Workflow for designing a DOE for NB generation**

The following provides a step-by-step workflow for designing the phase patterns on DOEs:

Step 1: Define key parameters for the NB

1. *Optical parameters of input laser beam and objective*

*2. The beam length (L) of the NB*

*3. The axial energy profile of the NB*

Step 2: Calculate the number of GB foci (M)

*1.* *Calculate M using the formular* $M=\frac{L}{RL} \sim\frac{L}{2RL}$*, where RL is the Rayleigh length of the objective. The average distance between two adjacent foci is approximately 0.5RL ~ RL.*

Step 3: Determine the positions of GB foci

*1. Initialize the position of the m^th^ GB focus (f_m_) as uniformly distributed along the length of NB*

*2. Iteratively adjust the intervals between consecutive GB foci to achieve the desired axial energy profile along each NB segment. The adjustment of an interval is proportional to the ratio between the simulated local intensity to the designed value. Specifically, if the simulated intensity is higher than the designed intensity, then the interval is increased to reduce the local intensity, if lower then decreased. It is expressed as* $Interval\left( m,i \right)=Interval\left( m,i-1 \right)\times{{Intensity}_{simulation}\left( m,i-1 \right)}/{{Intensity}_{design}\left( m,i-1 \right)}$*, where m stands for the m^th^ GB focus and i is the i^th^ iteration.*

Step 4 (single-plane DOE): Assign the binary matrix *L_m_(x,y)* with an equal number of pixels allocated for each GB focus, where (x, y) represents the coordinates on the DOE

Step 4 (dual-plane DOE): Assign the binary matrix *L_m_(x,y)* with different numbers of pixels allocated for GB foci located at the shallow and deep NB segments. The pixel allocation ratio between the two segments is set to 0.45:0.55, corresponding to an energy ratio of (0.44:0.55)^2^ = 0.4:0.6

Step 5: Define the phase regulator $P_{a_{m}}$

*1. Assign the phase regulator for the m^th^ GB focus as* $PA\times m$*, where PA is selected within the range [0, 2π]. Generally, the larger PA, the smaller the diameter of NB, also the lower efficiency of NB.*

Step 6: Generate and evaluate phase patterns on the DOE

*1. Create phase patterns by varying PA and axial positions of foci based on Equation 1 and 2 in the main text*

*2. Evaluate the generated NB using the following key metrics: beam diameter, energy in the main lobe, and the ratio of the 1^st^ side lobe to main lobe.*

*3. Select the optimal phase pattern based on the requirements for lateral resolution and energy efficiency.*

**Supplementary Note 2. Workflow for DOE fabrication**

The following outlines the step-by-step workflow for DOE fabrication:

| No. | Step | Tool | Process |
| --- | --- | --- | --- |
| 1 | Cleaning silica wafer | Wet bench | Soak blank fused silica wafers (4 inch, 500 μm thick) into piranha solution for 20 mins at 120 ℃. |
| 2 | Deposit 100 nm thick aluminum (Al) | Lesker Sputter | Set atmosphere pressure to 3 mT with downstream-DC-control. 750 s deposition for 100 nm Al onto the backside of a silica wafer. Al layer can give a better dry etching performance. |
| 3 | Coating photoresist | SVG Coat | Process the wafer with Hexamethyldisilazane for a better photoresist adhesion, then deposit 1.6 μm thick photoresist (SPR3612) on the wafer frontside. |
| 4 | Patterning | Heidelberg MLA 150 | Transfer a mask pattern onto the photoresist by the maskless direct write lithography tool, which uses a 405 nm laser with 1 defocus and 55 mJ/cm^2^ dosage. |
| 5 | Developing | SVG Developer | Bake the wafer at 110 °C for 90 s to harden the exposed photoresist then develop the patterned photoresist layer. |
| 6 | Dry Etching  (Inductively Coupled Plasma, ICP) | Plasma Therm Versaline | Pre-clean the chamber with O_2_ for 10 mins.  ICP parameters are set at 450 W ICP, 50 W BP, 40 CHF_3_, 2 O_2_, 5 mT, 4 T He, 10 °C electrode, 70 °C liner, 90 °C spool, 90 °C lip.  The etching rate is about 2.5 nm/s, which should be tested using a dummy wafer. Then etch a silica wafer with a corrected etching time. |
| 7 | Removing photoresist | Plasma Resist Strip, Matrix | Remove the remained photoresist by oxygen plasma. |
| 8 | Repeating |  | Repeat Steps 3-6 to complete the other three rounds of lithography. |
| 9 | Removing Al layer | Wet bench | Soak the wafer into Aluminum Etchant (CMOS, J.T. Baker) at 40 °C for 20 mins. |
| 10 | Dividing | DISCO Wafer Saw | Divide one wafer into separate six DOEs. |
